# Cross‐sex genetic correlations for fitness and fitness components: Connecting theoretical predictions to empirical patterns

**DOI:** 10.1002/evl3.116

**Published:** 2019-04-29

**Authors:** Tim Connallon, Genevieve Matthews

**Affiliations:** ^1^ School of Biological Sciences, and Centre for Geometric Biology Monash University Clayton Victoria 3800 Australia

**Keywords:** Additive genetic variance, genetic constraint, genetic trade‐off, sexual antagonism, sexual conflict

## Abstract

Sex differences in morphology, physiology, development, and behavior are widespread, yet the sexes inherit nearly identical genomes, causing most traits to exhibit strong and positive cross‐sex genetic correlations. In contrast to most other traits, estimates of cross‐sex genetic correlations for fitness and fitness components (rW fm ) are generally low and occasionally negative, implying that a substantial fraction of standing genetic variation for fitness might be sexually antagonistic (i.e., alleles benefitting one sex harm the other). Nevertheless, while low values of rW fm  are often regarded as consequences of sexually antagonistic selection, it remains unclear exactly how selection and variation in quantitative traits interact to determine the sign and magnitude of rW fm , making it difficult to relate empirical estimates of cross‐sex genetic correlations to the evolutionary processes that might shape them. We present simple univariate and multivariate quantitative genetic models that explicitly link patterns of sex‐specific selection and trait genetic variation to the cross‐sex genetic correlation for fitness. We show that rW fm  provides an unreliable signal of sexually antagonistic selection for two reasons. First, rW fm  is constrained to be less than the cross‐sex genetic correlation for traits affecting fitness, regardless of the nature of selection on the traits. Second, sexually antagonistic selection is an insufficient condition for generating negative cross‐sex genetic correlations for fitness. Instead, negative fitness correlations between the sexes (rW fm <0) can only emerge when selection is sexually antagonistic *and* the strength of directional selection on each sex is strong relative to the amount of shared additive genetic variation in female and male traits. These results imply that empirical tests of sexual antagonism that are based on estimates of rW fm  will be conservative and underestimate its true scope. In light of these theoretical results, we revisit current data on rW fm  and sex‐specific selection and find that they are consistent with the theory.

**Impact Summary**

Females and males differ, on average, for many traits, including size, shape, behavior, physiology, and lifespan. This sexual dimorphism is the outcome of an evolutionary history marked by sex differences in natural selection, where patterns of trait expression that are favored in one sex can be disfavored in the other (i.e., selection is “sexually antagonistic”). Although sexually antagonistic selection may favor the evolution of sex differences, it poses a problem for adaptation because strong genetic correlations between female and male traits limit the rate at which sex differences can evolve. This raises questions about whether sexually antagonistic selection is common in contemporary plant and animal populations, and how scientists might be able to empirically test whether it is. The cross‐sex genetic correlation for fitness (rW fm )—which quantifies the extent to which genotypes that are good for one sex are also good for the other—can serve as an empirical indicator of sexual antagonism, with negative estimates of rW fm  indicative of sexually antagonistic selection and pervasive genetic trade‐offs between female and male fitness. Several studies have estimated rW fm  for female and male fitness components, and found that rW fm  estimates are typically weakly positive, and occasionally negative. However, we lack formal theory allowing us to connect estimates of rW fm  to sex‐specific patterns of selection and genetic variation in quantitative traits, making rW fm  estimates difficult to interpret. We developed models showing that rW fm  is mathematically constrained to be weakly positive across a broad range of scenarios of selection, including cases where each sex has evolved to its optimum, as well as cases where selection is sexually antagonistic. Our results suggest that rW fm  is an unreliable proxy for sexual antagonism, and that direct estimates of phenotypic selection on each sex provide superior resolution concerning the pervasiveness of sexually antagonistic selection in nature.

As a rule, most genetic variation within a genome similarly affects the expression of female and male traits. For example, classical mutant alleles—including those identified by early *Drosophila* research—typically produce indistinguishable phenotypes when expressed by each sex (Ashburner et al. 2005). Chromosomes derived from mutation‐accumulation experiments, which carry combinations of random mutations, produce correlated patterns of sex‐specific variation in continuous traits such as wing size and shape (Houle and Fierst 2012). Likewise, the phenotypic effects of standing genetic variation in outbred animal and plant populations are nearly always positively correlated between the sexes (Lande 1980; Poissant et al. 2010; Griffin et al. 2013), and these positive genetic correlations are typically strong (Poissant et al. 2010).

Although most traits exhibit strong, positive genetic correlations between the sexes, there are two notable exceptions to this rule. First, genes affecting primary reproductive structures are much more likely to be sex‐limited in expression than genes affecting traits expressed by both sexes. For example, in *Drosophila*, sex‐limited genes tend to be expressed exclusively in the male‐limited testis or female‐limited ovaries (Meisel 2011). Sterility‐causing mutations also tend to be sex‐limited in expression (Lindsley and Lifschytz 1972), which partially shields them from purifying selection and elevates their equilibrium frequencies within a population (Connallon et al. 2010; Van Dyken and Wade 2010).

The second exception is the typically low cross‐sex genetic correlation for fitness and fitness components (e.g., survival, longevity, reproductive success). Fitness components generally exhibit much weaker genetic correlations between the sexes than most other traits (Poissant et al. 2010), with some estimates being negative (e.g, genotypes with relatively high fitness in one sex can have low fitness in the other; Brommer et al. 2007; Delcourt et al. 2009; Punzalan et al. 2014; Hendry et al. 2018). These low estimated genetic correlations for fitness and fitness components are believed to be hallmarks of sexually antagonistic selection (i.e., intralocus sexual conflict; Kruuk et al. 2008; Bonduriansky and Chenoweth 2009). Although traits affecting fitness are positively genetically correlated between the sexes, low fitness correlations arise as the relation between trait expression and fitness differs between the sexes (see Kruuk et al. 2008). The interaction between the shared genetic architecture of female and male traits and sex differences in selection on those traits can either dampen an otherwise positive genetic correlation for fitness between the sexes, or cause intersexual fitness correlations to become negative.

How much must selection differ between the sexes for cross‐sex genetic correlations for fitness to become negative? More generally, how exactly do patterns of sex‐specific selection and genetic variation in female and male traits interact to shape the cross‐sex genetic correlation for fitness? Prior theory has considered the effects of sex differences in selection on the distribution of sex‐specific fitness effects among new mutations (Connallon and Clark 2014), and the impact of sexual conflict over resource allocation between fitness components on the fitness covariance between the sexes (Zajitschek and Connallon 2017). However, there is currently no general theoretical prediction that explicitly links sex‐specific patterns of selection on quantitative traits and standing genetic variation within those traits to the genetic correlation between female and male fitness. Such theory should prove useful for interpreting empirical estimates of cross‐sex genetic correlations for fitness or fitness components (e.g., Poissant et al. 2010; Duffy et al. 2014; Punzalan et al. 2014; reviewed in the Discussion), and evaluating evidence for sexually antagonistic selection and sexually antagonistic genetic variation from dioecious plant and gonochoristic animal populations.

Here, we present simple models of sex‐specific fitness variance and covariance between the sexes, in which the fitness of individuals from each sex depends on the expression of one or more quantitative traits under selection to an optimum. We use the models to derive mathematical relations between sex‐specific selection, sex‐specific trait variances, and the cross‐sex genetic correlation for fitness. The models reveal simple, yet counterintuitive, criteria for positive versus negative cross‐sex fitness correlations. We show that cross‐sex genetic correlations for fitness exhibit a bias toward weakly positive values, regardless of whether or not selection is sexually antagonistic (i.e., selection in opposing directions between the sexes). Negative fitness correlations between the sexes arise when selection is sexually antagonistic *and* the strength of directional selection in each sex is strong relative to the amount of shared additive genetic variation in traits underlying fitness. Our results imply that genetic correlations for fitness may often be positive in the presence of unresolved sexually antagonistic selection, and that negative cross‐sex genetic correlations for fitness represent conservative evidence for sexually antagonistic variation.

## Fitness as a Function of Single Traits

We begin by presenting the simplest version of our model, in which fitness variation within each sex depends on the expression of a single quantitative trait under selection to sex‐specific optima for the trait. In an attempt to promote accessibility of the theory, we provide a very detailed derivation of the model, with each step of the derivation outlined in Appendix I of the Supporting Information.

We assume that the additive genetic variance in the trait is composed of shared and sex‐limited components. For example, the trait's breeding value for an individual of the *j*th sex is *z_j_* = *x* + *y_j_*, where *x* represents the shared genetic component of the trait and *y_j_* represents the sex‐limited component for that sex (*j*∈{*f*, *m*}, with subscripts referring to female and male, respectively); *x*, *y_f_*, and *y_m_* are assumed to be independent, normally distributed random variables. Fitness for members of the *j*th sex follows a Gaussian function of trait expression in that sex:

(1) $$W_{j}\propto \exp\left(-\frac{{\left(O_{j}-z_{j}\right)}^{2}}{2\omega_{j}^{2}}\right)$$

where *O_j_* is the optimum of the *j*th sex, and *ω_j_* is a constant describing the rate of fitness decline away from the optimum. Since we are exclusively concerned with additive genetic variances, covariances, and correlations, we will ignore effects of random environmental variation on trait expression, although the inclusion of such factors will not alter our conclusions. For mathematical convenience and simplicity, we present exact results for fitness variance and covariance on logarithmic scale. Results in standard scale (i.e., not log‐scaled) closely match those of log‐scale fitness, particularly in the biologically relevant scenario of a population that is reasonably well adapted to its environment (discussed in Lande 1976; Manna et al. 2011). See Appendix I of the Supporting Information for further discussion and a direct comparison of results for both fitness scales.

To quantify sex‐specific variances and between‐sex covariances for traits and fitness, assume that $\sigma_{x}^{2}$ is the shared additive genetic variance and $\sigma_{y_{f}}^{2}$ and $\sigma_{y_{m}}^{2}$ are sex‐limited additive genetic variances for the trait; the overall additive genetic variances for females and males are $\sigma_{f}^{2}=\sigma_{x}^{2}+\sigma_{y_{f}}^{2}$ and $\sigma_{m}^{2}=\sigma_{x}^{2}+\sigma_{y_{m}}^{2}$. Sex‐specific fitness variance and between‐sex fitness covariance are:

(2) var lnWj=σj22dj2+σj22ωj4

and

(3) cov lnWf,lnWm=σx22dfdm+σx22ωf2ωm2

where $d_{j}=O_{j}-{\bar{z}}_{j}$ is the displacement, for the *j*th sex, of the trait mean from the optimum (the overbar denotes the average of *z_j_*). The cross‐sex additive genetic correlation for fitness is:

(4) rW fm =rz2dfdm+rzσfσm2df2+σf22dm2+σm2

where $r_{z}=\sigma_{x}^{2}{\left(\sigma_{f}\sigma_{m}\right)}^{-1}$ is the genetic correlation for the trait. Notably, the cross‐sex genetic correlation for fitness is insensitive to details of fitness surface curvature (i.e., $\omega_{f}^{2}$ and $\omega_{m}^{2}$), and instead reduces to a remarkably simple function of the sex‐specific additive genetic variances ($\sigma_{f}^{2}$, $\sigma_{m}^{2}$), the cross‐sex genetic correlation for the trait ($r_{z}$), and the displacement of each sex from its optimum ($d_{f}=O_{f}-{\bar{z}}_{f}$, $d_{m}=O_{m}-{\bar{z}}_{m}$).

**Figure 1:**
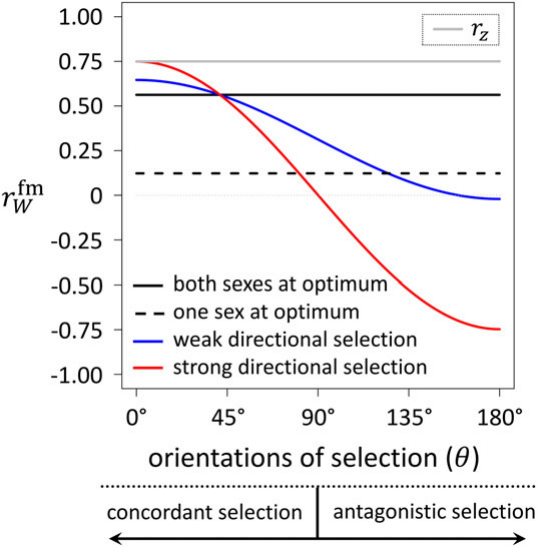
**Impact of sex‐specific directional selection on the cross‐sex genetic correlation for fitness (** $r_{W}^{\mathrm{fm}}$ **)**. Four scenarios of sex‐specific selection are illustrated: (1) both sexes are at their optima ($d_{f}=d_{m}=0$), (2) one sex is at optimum and the other is modestly displaced ($d_{f}=0;\;d_{m}=n\sigma_{x}^{2}$), (3) both sexes are weakly displaced from their optima ($d_{f}=d_{m}=n\sigma_{x}^{2}/5$), and (4) both sexes are strongly displaced from their optima ($d_{f}=d_{m}=5n\sigma_{x}^{2}$). The orientations of directional selection in each are perfectly aligned when *θ* = 0°, and perfectly opposing when *θ* = 180°. Selection largely aligns between the sexes (it is “sexually concordant”) when 0° ≤ *θ* < 90°; selection is largely sexually antagonistic when 90° ≤ *θ* ≤ 180°. Results, which are based on equation (11), assume an empirically representative cross‐sex genetic correlation of $r_{z}=0.75$ for the quantitative traits (displayed in gray).

Two key insights emerge from equation (4). First, the genetic correlation for fitness is constrained to be less than the genetic correlation for the trait, regardless of the specific form of selection in each sex (i.e., rW fm <rz, provided 0 < $r_{z}$ < 1, as current data strongly support; see Poissant et al. 2010). The theoretical maximum of rW fm  occurs when both sexes are strongly displaced from their optima and directional selection is aligned between the sexes (i.e., rW fm ≈rz when *d_f_d_m_* ≫ $r_{z}\sigma_{f}\sigma_{m}$, *d_j_* ≫ $\sigma_{j}$). Such a scenario might occur immediately following a change in environment that generates concordant directional selection between the sexes, leading to parallel short‐term evolutionary changes in female and male traits (see Lande 1980; Connallon and Hall 2016). All other scenarios of selection lead to a reduction in rW fm  relative to $r_{z}$. A special case arises when both sexes are at optimum (*d_f_* = *d_m_* = 0, which represents the long‐term evolutionary equilibrium for the population; Lande 1980), wherein equation (4) reduces to rW fm =rz2. Consequently, rW fm  will always be weaker than $r_{z}$ —and considerably weaker when trait genetic correlations are modest in magnitude (e.g., rW fm ≤rz/2 when $0<r_{z}\leq 1/2$)—in well‐adapted populations in which sexual antagonism has been completely resolved (see Fig. 1 for a relevant example).

Second, equation (4) implies that conditions for a negative genetic correlation for fitness are restrictive, and that rW fm  can be positive even in the presence of sexually antagonistic selection. Assuming $r_{z}>0$, the criterion for a negative genetic correlation for fitness (rW fm <0) is:

(5) $$d_{f}d_{m}<-\frac{1}{2}\sigma_{x}^{2}$$

and otherwise rW fm  will be positive (note that the inequality can also be expressed as $d_{f}d_{m}<-\frac{1}{2}r_{z}\sigma_{f}\sigma_{m}$). Negative rW fm  requires both sexually antagonistic selection (one sex below optimum and the other sex above optimum: $d_{f}d_{m}<0$) *and* large displacements of each sex from its optimum relative to the amount of shared genetic variance in the trait (i.e., $|d_{f}d_{m}|$ must be greater than $\sigma_{x}^{2}/2$). Equation (5) can also be expressed as a function of sex‐specific linear selection gradients, *β_f_* and *β_m_*, which are widely estimated in animal populations (Cox and Calsbeek 2009; Morrissey 2016; De Lisle et al. 2018; Singh and Punzalan 2018). Provided the trait is genetically variable ($\sigma_{f},\sigma_{m}>0$), the criterion for rW fm <0becomes:

(6) $$\beta_{f}\beta_{m}<-\frac{1}{2}\sigma_{x}^{2}\gamma_{f}\gamma_{m}$$

where *γ_f_* and *γ_m_* represent the strengths of stabilizing selection on each sex ($\beta_{f}=\gamma_{f}d_{f}$ and $\beta_{m}=\gamma_{m}d_{m}$). From equation (6), we see that selection must be sexually antagonistic ($\beta_{f}\beta_{m}<0$) and directional selection on each sex must be sufficiently strong for the genetic correlation for fitness to be negative.

## Fitness as a Function of Multivariate Traits

Conclusions from the single trait model also apply to contexts of multivariate selection. We consider an idealized scenario involving *n* traits that vary independently of one another and contribute independently to fitness (i.e., correlational selection and genetic correlations between traits are negligible). As before, the phenotype is affected by shared and sex‐limited additive genetic variance for each trait. The breeding value for the *i*th trait expressed in the *j*th sex is *z_i,j_* = *x_i_* + *y_i,j_*, where *x_i_* is the shared genetic variance and *y_i,j_* is the sex‐limited variance of the trait; *x_i_*, *y_i,f_*, and *y_i,m_* are once again assumed to be independent, normally distributed random variables. The additive genetic variance for the *i*th trait in the *j*th sex is $\sigma_{i,j}^{2}=\sigma_{i,x}^{2}+\sigma_{i,y_{j}}^{2}$, where $\sigma_{i,x}^{2}$ and $\sigma_{i,y_{j}}^{2}$ represent the shared and sex‐limited components of trait variation; the genetic correlation for the trait is $r_{i,z}=\sigma_{i,x}^{2}{\left(\sigma_{i,f}\sigma_{i,m}\right)}^{-1}$.

Fitness in the *j*th sex is a function of the set of trait displacements from the multivariate trait optimum:

(7) $$W_{j}\propto \exp\left(-\frac{1}{2}\sum_{i=1}^{n}\frac{{\left(O_{i,j}-z_{i,j}\right)}^{2}}{\omega_{i,j}^{2}}\right)$$

where $O_{i,j}$ is the optimum, and $\omega_{i,j}^{2}$ is the fitness surface curvature, for the *i*th trait of the *j*th sex. The genetic variances and cross‐sex genetic covariance for fitness, expressed on log scale, are:

(8) var lnWj=12∑i=1nσi,j22di,j2+σi,j2ωi,j4

and

(9) cov lnWf,lnWm=12∑i=1nσi,x22di,fdi,m+σi,x2ωi,f2ωi,m2

where $d_{i,j}=O_{i,j}-{\bar{z}}_{i,j}$ (see Appendix II of the Supporting Information). The cross‐sex additive genetic correlation for fitness becomes:

(10) rW fm =∑i=1nσi,x22di,fdi,m+σi,x2ωi,f2ωi,m2∑i=1nσi,f22di,f2+σi,f2ωi,f4∑i=1nσi,m22di,m2+σi,m2ωi,m4

The general expression for rW fm  is considerably more complicated than that of the univariate model (eq. (4)). To build intuition, we first explore its behavior for an idealized scenario, in which the genetic variance and strength of stabilizing selection is constant across traits ($\sigma_{j}=\sigma_{i,j}$ and $\omega_{j}=\omega_{i,j}$; this scenario is analogous to the classic (isotropic) version of Fisher's Geometric Model; see Waxman and Welch 2005; Martin and Lenormand 2006; Martin 2014; Tenaillon 2014). For this scenario, equation (10) simplifies to:

(11) rW fm =rz2dfdmcosθ+nrzσfσm2df2+nσf22dm2+nσm2

where $d_{j}=\sqrt{\sum_{i=1}^{n}d_{i,j}^{2}}$ is the total displacement of the *j*th sex from its optimum (the Euclidean norm of the vector of displacements), θ is the angle between the vectors of displacement, and $\cos\left(\theta \right)={\left(d_{f}d_{m}\right)}^{-1}\sum_{i=1}^{n}d_{i,f}d_{i,m}$ is a measure of the correlation between the set of female and male trait displacements (see Connallon and Clark 2014).

Equation (11) reinforces the major conclusions from the univariate model (Fig. 1). As before, $r_{z}$ provides an upper limit for rW fm , with rW fm ≈rz when both sexes are strongly displaced from their optima and selection is perfectly aligned between them (Fig. 1, red curve at *θ* = 0); other scenarios of selection reduce rW fm  relative to $r_{z}$. Likewise, sexually antagonistic selection remains an insufficient condition for generating a negative cross‐sex genetic correlation for fitness. Assuming $r_{z}$ is positive, we see that rW fm  (from eq. (11)) will be negative if and only if:

(12) $$\cos\left(\theta \right)<-\frac{n\sigma_{x}^{2}}{2d_{f}d_{m}}$$

There are two requirements for this condition to be met. First, the angle between the sex‐specific vectors of displacement must exceed orthogonality (i.e., cos(*θ*) < 0 requires that 90° < *θ* ≤ 180°), which is a conservative criterion for sexually antagonistic selection on the set of traits (see Connallon and Hall 2018). Second, the magnitude of the displacement of each sex from its optimum ($d_{f}$ and $d_{m}$) must be large relative to the magnitude of the shared genetic variance in the set of traits (i.e., $n\sigma_{x}^{2}$). Since the minimum possible value of cos(*θ*) is –1, the genetic correlation for fitness cannot be negative unless $n\sigma_{x}^{2}<2d_{f}d_{m}$ (e.g., the red curve in Fig. 1, in which rW fm  is negative when *θ* > ∼90°). Consequently, when the sexes are weakly displaced from their optima, or at least one sex is at optimum, fitness will always be positively correlated between the sexes, with or without sexually antagonistic selection (see in Fig. 1, e.g., the blue curve and the dashed black line, in which 1 ≫ rW fm >0 in spite of unresolved sexual antagonism in both instances).

Qualitatively similar results emerge if we permit traits to differ in the amount of standing genetic variation and/or the strength of stabilizing selection (see Appendix II of the Supporting Information). For example, consider a well‐adapted population, in which the trait means of each sex match the set of optima ($d_{i,j}=0$), and cross‐sex genetic correlations are constant across traits ($r_{z}=r_{i,z}$). In this scenario, equation (10) simplifies to:

(13) rW fm =rz2cosθ0

where θ_0_ is the angle between sex‐specific vectors $I_{0,f}=\frac{1}{2}\left(\sigma_{1,f}^{2}\omega_{1,f}^{-2},\sigma_{2,f}^{2}\omega_{2,f}^{-2},\ldots ,\sigma_{n,f}^{2}\omega_{n,f}^{-2}\right)$ and $I_{0,m}=\frac{1}{2}\left(\sigma_{1,m}^{2}\omega_{1,m}^{-2},\sigma_{2,m}^{2}\omega_{2,m}^{-2},\ldots ,\sigma_{n,m}^{2}\omega_{n,m}^{-2}\right)$. In equation (13), $\cos(\theta_{0})$ describes the alignment between the sexes for the standing genetic load for the set of traits. When elements of the load for each sex have the same proportionality ($\sigma_{i,f}^{2}\omega_{i,f}^{-2}=c\sigma_{i,m}^{2}\omega_{i,m}^{-2}$, where *c* is a constant), then $\cos(\theta_{0})=1$ and rW fm =rz2, as before. Otherwise, $\cos(\theta_{0})<1$ and rW fm  is further depressed relative to $r_{z}$.

In a poorly adapted population in which both sexes are strongly displaced from their optima ($|d_{i,f}d_{i,m}|$ ≫ $\sigma_{i,x}^{2}$, $|d_{i,j}|$ ≫ $\sigma_{i,j}$), and cross‐sex genetic correlations are constant across traits ($r_{z}=r_{i,z}$), equation (10) reduces to:

(14) rW fm =rzcosθd

where $\theta_{d}$ is the angle between sex‐specific vectors $I_{d,f}=\left(\sigma_{1,f}d_{1,f}\omega_{1,f}^{-2},\sigma_{2,f}d_{2,f}\omega_{2,f}^{-2},\ldots ,\sigma_{n,f}d_{n,f}\omega_{n,f}^{-2}\right)$ and $I_{d,m}=\left(\sigma_{1,m}d_{1,m}\omega_{1,m}^{-2},\sigma_{2,m}d_{2,m}\omega_{2,m}^{-2},\ldots ,\sigma_{n,m}d_{n,m}\omega_{n,m}^{-2}\right)$. Note that ***I***_***d***, ***j***_ is roughly equivalent to the set of variance‐weighted linear selection gradients for the *j*th sex; under weak stabilizing selection, $\sigma_{i,j}d_{i,j}\omega_{i,j}^{-2}\approx \sigma_{i,j}\beta_{i,j}$, where $\beta_{i,j}\approx d_{i,j}\omega_{i,j}^{-2}$ is the linear selection gradient for the *i*th trait in the *j*th sex. In equation (14), $\cos(\theta_{d})$ describes the alignment between sexes in directional selection, taking into account the amount of standing genetic variation in each of the traits. As before, sexually antagonistic selection can cause rW fm  to become negative when directional selection differs strongly between the sexes; this possibility becomes particularly likely when traits subject to sexually antagonistic selection have relatively high standing genetic variation compared to traits where female and male selection is in the same direction (e.g., traits with negative $d_{i,f}d_{i,m}$ have high $\sigma_{i,j}^{2}$ relative to traits with positive $d_{i,f}d_{i,m}$).

## Discussion

### QUALITATIVE PREDICTIONS OF THE THEORY

Our theory yields two key predictions that are pertinent to empirical estimates of sex‐specific selection and of cross‐sex genetic correlations for quantitative traits and for fitness. First, cross‐sex genetic correlations for fitness (rW fm ) will always be weaker than genetic correlations between male and female traits that mediate fitness ($r_{z}$), regardless of the specific form of selection on each sex. This prediction applies broadly, including cases in which selection is sexually antagonistic, as well as cases in which both sexes are at optimum and all genetic variation has sexually concordant fitness effects (e.g., Connallon and Clark 2014). The empirical observation that cross‐sex genetic correlations for fitness components are weaker than cross‐sex genetic correlations for other traits (see Poissant et al. 2010) is consistent with this prediction.

Our second prediction is that genetic correlations for fitness are constrained to be positive under a broad range of scenarios of sex‐specific selection, including some scenarios in which selection acts in opposite directions between the sexes. Negative genetic correlations require both sexually antagonistic selection on traits affecting fitness *and* sufficiently pronounced maladaptation in both sexes relative to the standing genetic variation in traits mediating fitness. Interestingly, population genetics theory predicts that sexually antagonistic selection should, if anything, elevate standing genetic variation at loci expressed by both sexes (e.g., Kidwell et al. 1977; reviewed in Connallon and Chenoweth 2019), which raises the paradoxical possibility that high standing genetic variation, resulting from a history of sexually antagonistic selection, may end up obscuring empirical signals of fitness trade‐offs between the sexes, as manifest in a negative rW fm .

Overall, our predictions suggest that sexually antagonistic genetic variation does not necessarily need to be invoked to account for most estimates of rW fm , which are weakly positive. While, sexual antagonism can, and probably does, play some role in reducing rW fm  relative to $r_{z}$, estimates of rW fm  do not, by themselves, allow us to evaluate whether a population is currently experiencing sexually antagonistic selection. The one exception—those rare estimates of rW fm  that are significantly negative (see Table 1), and which only make sense in light of strong sexually antagonistic selection—provide conservative evidence of ongoing sexual antagonism.

**Table 1:** A survey of sex‐specific selection estimates and cross‐sex genetic correlations for fitness components. Sex‐specific selection data were compiled by Cox and Calsbeek (2009). Cross‐sex genetic correlation data were compiled from an extensive literature search and are presented in Table S1

A. Sex‐specific directional selection gradients				
	***n***	***β_f_β_m_* < 0**	***β_f_β_m_* > 0**	**Pr(*β_f_β_m_* < 0)**
**All estimates** *	203	78	125	0.38
**Viability** * ^,^ †	71	24	47	0.34
**Reproductive success** * ^,^ ‡	132	54	78	0.41
**Significant estimates** §	33	13	20	0.39

B. Cross‐sex genetic correlations for fitness components (rW fm )				
	***n***	$r_{W}^{\mathrm{fm}}$ **< 0**	$r_{W}^{\mathrm{fm}}$ **> 0**	**Pr(** $r_{W}^{\mathrm{fm}}$ **< 0)**
**All estimates** *	86	28	58	0.33
**Viability** * ^,^ †	39	5	34	0.13
**Reproductive success** * ^,^ ‡	47	23	24	0.49
**Significant estimates** §	29	6	23	0.21

^*^Selection gradient estimates are from Appendix B of Cox and Calsbeek (2009), which controls for spatial and temporal replication within studies; rW fm  estimates are from Table S1 (current study).

^†^Selection gradients and cross‐sex genetic correlations related to juvenile or adult viability.

^‡^Selection gradients and cross‐sex genetic correlations related to reproductive success, including sexual selection, fecundity selection, and net selection or overall fitness.

^§^Estimates that were: (1) statistically significant for directional selection in both sexes (from Appendix A of Cox and Calsbeek 2009); or (2) significantly different from rW fm  = 0 (Table S1 of the current study; among the multiple proxies of fitness used in Foerster et al. (2007) we used the rW fm  estimate for their metric, “contribution to population growth”).

Why exactly are fitness correlations between sexes constrained to be positive as both sexes approach their optima? Why is it easier for rW fm  to be negative when both sexes are far from optimum? The sign of rW fm  reflects a balance between two sources of sex‐specific maladaptation, each affecting the expression of rW fm  in different ways. Genetic load within each sex can be partitioned into a “lag load,” caused by deviation between the average trait and the optimal trait, and a standing genetic load caused by phenotypic variation around the mean (see Lande and Shannon 1996; Kirkpatrick and Barton 1997; Chevin 2013). When both sexes are far from optimum, maladaptation is primarily attributable to the lag load, and the sign of rW fm  is determined by the alignment (or misalignment) between directional selection in each sex. However, as each sex approaches its optimum, maladaptation is increasingly attributable to the standing genetic load; deviations from the population mean become increasingly maladaptive, and in this case, rW fm  increases with the cross‐sex genetic correlation for traits affecting fitness and becomes insensitive to the sex‐specific orientations of directional selection. The conflicting effects of lag and variance loads on rW fm  are qualitatively similar to the opposing effects of different sources of variation, during development, on genetic correlations between traits that trade‐off against one another. For example, the opposing effects of variation in resource acquisition relative to variation in resource allocation can mask empirical signals of trade‐offs between different fitness components that are expressed by members of a single sex (e.g., survival vs. fecundity; see van Noordwijk and de Jong 1986; Metcalf 2016), as well as signals of trade‐offs between the sexes (Zajitschek and Connallon 2017).

### EFFECTS OF ADAPTATION AND ENVIRONMENTAL CHANGE ON $r_{W}^{\mathrm{fm}}$

Our results also clarify how processes of adaptation and environmental change are likely to influence fitness correlations between the sexes—a subject of much recent attention (Long et al. 2012; Berger et al. 2014; Punzalan et al. 2014; Han and Dingemanse 2017; Holman and Jacomb 2017; Martinossi‐Allibert et al. 2018; reviewed in Connallon and Hall 2018). The process of adaptation tends to bring both sexes closer to their optima, while simultaneously increasing sex differences in the orientation of directional selection (see Lande 1980; Connallon and Clark 2014; Connallon and Hall 2018; in the context of our model, adaptation is expected to increase θ and decrease $d_{f}$ and $d_{m}$). The net effect of these two types of changes on rW fm  depends upon the tempo with which θ increases (which should decrease rW fm ) relative to the tempo with which $d_{f}$ and $d_{m}$ decrease (which will elevate rW fm  if selection is sexually antagonistic; *cf*. red vs. blue curves in Fig. 1). Thus, although the sex‐specific directions of selection are expected to become increasingly sexually antagonistic during adaptation toward stationary female and male optima (Connallon and Hall 2018), conditions for a negative rW fm  may not necessarily become more permissive during this process.

Environmental change is expected to reduce population adaptation, and thereby alter genetic correlations between female and male fitness. The key to whether such changes increase or decrease rW fm  is whether directional selection in novel environments tends to be aligned between the sexes. If, as suggested by some studies, novel environments tend to reduce sexual conflict (Berger et al. 2014; Connallon and Hall 2016; but see Holman and Jacomb 2017; Martinossi‐Allibert et al. 2018), then rW fm  should typically increase following environmental change. These consequences of environmental change for rW fm  can be further modified by environmentally induced changes in the expression of quantitative genetic variation in addition to changes in selection. For example, exposure to new developmental conditions can induce expression of “cryptic” genetic variation that is masked in ancestral environments (McGuigan and Sgrò 2009) or alter trait genetic correlations (Sgrò and Hoffmann 2004; Wood and Brodie 2015). Few studies have addressed the potential effects of environmental change on sex‐specific variances and cross‐sex genetic correlations in quantitative traits, although such effects could have significant consequences for rW fm  and the expression of sexual dimorphism in changing environments (see Ketola et al. 2012).

### RELATION BETWEEN THEORY AND EMPIRICAL DATA ON $r_{W}^{\mathrm{fm}}$ AND SEX‐SPECIFIC SELECTION

Two types of data have been leveraged for testing hypotheses about sexually antagonistic selection. A substantial number of studies have estimated additive genetic correlations between sexes for fitness components (Poissant et al. 2010; Table S1), with negative correlations between the sexes taken as unambiguous signals of sexual conflict. Second, an even larger number of studies have published empirical estimates of female‐ and male‐specific selection on homologous traits that both sexes express (Cox and Calsbeek 2009; Morrissey 2016; De Lisle et al. 2018; Singh and Punzalan 2018). These data provide clear evidence of sexually antagonistic selection in cases where female and male selection gradients of a trait exhibit opposite signs (*β_f_β_m_* < 0; e.g., Cox and Calsbeek 2009; Morrissey 2016; Sanjaka et al. 2017), or when the angle between multivariate vectors of sex‐specific selection gradients is greater than orthogonal (i.e., θ > 90°; Lewis et al. 2011; Gosden et al. 2012; Stearns et al. 2012). Note that sexually antagonistic genetic variation should be present under a broader set of conditions, as even minor misalignment between female and male directional selection (i.e., *θ* > 0°) should cause a fraction of standing genetic variation to have sexually antagonistic fitness effects (Connallon and Clark 2014).

Our theory suggests that empirical examples of traits subject to sexually antagonistic selection should be more common than examples of negative fitness correlations between the sexes. Logistics of experimental tractability and statistical power clearly play a role in the types of population parameters that are most readily estimated for a given population, as well as the precision of these estimates. Aspects of quantitative genetics experimental designs, including whether assayed individuals come from controlled lab crosses or field‐sampled individuals from pedigreed populations, can also impact inferences of fitness variance and variance in adult fitness components that are closely tied to the species’ mating system (see Reid 2014). However, beyond such logistical considerations, our theory shows that the criterion for sexually antagonistic selection on phenotypes (e.g., *β_f_β_m_* < 0 for univariate traits) is always easier to meet than the criterion for a negative cross‐sex correlation for fitness (rW fm  < 0). Similar predictions apply to selection on multivariate traits.

A summary of estimates of univariate sex‐specific selection and cross‐sex fitness correlations is consistent with our theoretical predictions (Table 1, based upon published point estimates of $\beta_{f}$, $\beta_{m}$, and rW fm ). The fraction of traits subject to sexually antagonistic selection is higher than the fraction of rW fm  estimates that is negative. This pattern also holds if we focus on statistically significant estimates of directional selection and cross‐sex correlations for fitness components (Table 1). It should be kept in mind that selection gradient estimates typically lack precision, and noise in the estimates are likely to upwardly bias the proportion of traits exhibiting selection gradient estimates with opposite signs between sexes (Morrissey 2016). For example, Morrissey (2016) showed that accounting for uncertainty in selection gradient estimates reduces the estimated fraction of sexually antagonistic traits from 40% (based on point estimates of *β_f_* and *β_m_*) to roughly 20% (based on simulations that assume *β_f_* and *β_m_* follow a bivariate normal distribution among traits). Similar concerns also apply to rW fm  point estimates, which have notoriously large confidence intervals that typically overlap with zero. Publication bias toward negative estimates of rW fm  might also drive up the proportion of estimates that is negative. We found six studies reporting significantly negative estimates of rW fm  for fitness or a fitness component (Table 1). The significant result from one of these studies was highly sensitive to the analysis used to analyze rW fm  (see Walling et al. 2014); four of the remaining studies were on lab populations of *Drosophila*. Overall, clear examples of negative rW fm  estimates are rare. This rarity may reflect the difficulty of accurately estimating rW fm  as well as the possibility that sexually antagonistic selection, when present, occurs in reasonably well‐adapted populations where the strength of directional selection is modest‐to‐weak (see Morrissey 2016).

Associate Editor: A. Charmantier

## Supporting information

Supporting Information

Supporting Information
