# Supplementary material for: Cross‐sex genetic correlations for fitness and fitness components: Connecting theoretical predictions to empirical patterns
Source: Evol Lett. 2019 Apr 29;3(3):254–62. doi: 10.1002/evl3.116 (PMC6546386; doi:10.1002/evl3.116)
Supplement: Supplementary file 2 — Supporting Information [file EVL3-3-254-s002.pdf]

Appendix I. A detailed derivation of the univariate model

Assuming that fitness follows a Gaussian function of trait expression, the sex-specific fitness variances and the cross-sex genetic covariance for fitness can be calculated from the following general expression for fitness in members of the *j*th sex:

$$W_j = C_j \exp\left(-\frac{\left(O_j - z_j\right)^2}{2\omega_j^2}\right) \qquad \text{(A1)},$$

where C_j is an arbitrary, positive constant that defines the maximum of the fitness function.

Logarithmic scale calculations for Gaussian fitness functions

On a logarithmic scale, where $\ln\left(W_j\right) = \ln\left(C_j\right) - \frac{\left(O_j - z_j\right)^2}{2\omega_j^2}$, calculations of sex-specific fitness variances and cross-sex genetic correlation for fitness are straightforward. The variance within the *j*th sex is calculated as follows:

$$\operatorname{var}\left[\ln\left(W_j\right)\right] = \operatorname{var}\left[\ln\left(C_j\right) - \frac{\left(O_j - z_j\right)^2}{2\omega_j^2}\right] = \operatorname{var}\left[\frac{\left(O_j - z_j\right)^2}{2\omega_j^2}\right] \qquad \text{(A2)},$$

$$= E\left[\frac{\left(O_j - z_j\right)^4}{4\omega_j^4}\right] - \left(E\left[\frac{\left(O_j - z_j\right)^2}{2\omega_j^2}\right]\right)^2$$

where E[] refers to the expectation of the function within the square brackets. Eq. (A2) follows from the general definition of variance of a random variable, *X*: $\operatorname{var}(X) = E[(X - \bar{X})^2] = E[X^2 - 2X\bar{X} + \bar{X}^2] = E[X^2] - \bar{X}^2$, where the overbar is shorthand for the expectation. Other aspects of the calculation follow from basic probability theory (*e.g.*, https://en.wikipedia.org/wiki/Variance#Properties).

The terms of the final expression in eq. (A2) can be calculated as follows:

$$E\left[\frac{\left(O_j - z_j\right)^4}{4\omega_j^4}\right] = \frac{E\left[O_j^4 - 4O_j^3 z_j + 6O_j^2 z_j^2 - 4O_j z_j^3 + z_j^4\right]}{4\omega_j^4}$$

$$= \frac{O_j^4 - 4O_j^3 E\left[z_j\right] + 6O_j^2 E\left[z_j^2\right] - 4O_j E\left[z_j^3\right] + E\left[z_j^4\right]}{4\omega_j^4}$$

$$= \frac{O_j^4 - 4O_j^3 \bar{z}_j + 6O_j^2\left(\bar{z}_j^2 + \sigma_j^2\right) - 4O_j\left(\bar{z}_j^3 + 3\bar{z}_j\sigma_j^2\right) + \bar{z}_j^4 + 6\bar{z}_j^2\sigma_j^2 + 3\sigma_j^4}{4\omega_j^4}$$

where $E\left[z_j\right] = \bar{z}_j$, $E\left[z_j^2\right] = \bar{z}_j^2 + \sigma_j^2$, $E\left[z_j^3\right] = \bar{z}_j^3 + 3\bar{z}_j\sigma_j^2$, and $E\left[z_j^4\right] = \bar{z}_j^4 + 6\bar{z}_j^2\sigma_j^2 + 3\sigma_j^4$, and:

$$\left(E\left[\frac{\left(O_j - z_j\right)^2}{2\omega_j^2}\right]\right)^2 = \left(\frac{E\left[O_j^2 - 2O_j z_j + z_j^2\right]}{2\omega_j^2}\right)^2 = \frac{\left(\left(O_j - \bar{z}_j\right)^2 + \sigma_j^2\right)^2}{4\omega_j^4}$$

Substituting these expressions back into eq. (A2), and following some algebra, the variance of the *j*th sex eventually simplifies to:

$$\mathrm{var}\left[\ln\left(W_j\right)\right] = \sigma_j^2 \frac{2\left(O_j - \bar{z}_j\right)^2 + \sigma_j^2}{2\omega_j^4} \quad \text{(A3)},$$

which is equivalent to eq. (2) in the main text.

The cross-sex covariance is calculated as:

$$\mathrm{cov}\left[\ln\left(W_f\right), \ln\left(W_m\right)\right] = \mathrm{cov}\left[\ln\left(C_f\right) - \frac{\left(O_f - z_f\right)^2}{2\omega_f^2}, \ln\left(C_f\right) - \frac{\left(O_m - z_m\right)^2}{2\omega_m^2}\right] \quad \text{(A4)},$$

$$= \mathrm{cov}\left[\frac{\left(O_f - z_f\right)^2}{2\omega_f^2}, \frac{\left(O_m - z_m\right)^2}{2\omega_m^2}\right] = \frac{\mathrm{cov}\left[-2O_f z_f + z_f^2, -2O_m z_m + z_m^2\right]}{4\omega_f^2\omega_m^2}$$

$$= \frac{4O_f O_m \mathrm{cov}\left[z_f, z_m\right] - 2O_f \mathrm{cov}\left[z_f, z_m^2\right] - 2O_m \mathrm{cov}\left[z_f^2, z_m\right] + \mathrm{cov}\left[z_f^2, z_m^2\right]}{4\omega_f^2\omega_m^2}$$

Recalling that x, y_f and y_m are independently distributed random variables, with $\bar{z}_j = E\left[z_j\right] = E[x] + E\left[y_j\right]$, the covariance terms in the final expression of eq. (A4) can be calculated as follows:

$$\mathrm{cov}\left[z_f, z_m\right] = \mathrm{cov}\left[x + y_f, x + y_m\right] = \sigma_x^2$$

$$\mathrm{cov}[z_f, z_m^2] = \mathrm{cov}[x + y_f, x^2 + 2xy_m + y_m^2] = \mathrm{cov}[x, x^2] + 2\mathrm{cov}[x, xy_m]$$

$$= (E[x^3] - E[x^2]E[x]) + 2(E[x^2 y_m] - E[x]E[xy_m])$$

$$= 2E[x]\sigma_x^2 + 2E[y_m]\sigma_x^2 = 2\bar{z}_m \sigma_x^2$$

$$\mathrm{cov}[z_f^2, z_m] = \mathrm{cov}[x^2 + 2xy_f + y_f^2, x + y_m] = \mathrm{cov}[x^2, x] + 2\mathrm{cov}[xy_f, x]$$

$$= (E[x^3] - E[x^2]E[x]) + 2(E[x^2 y_f] - E[xy_f]E[x])$$

$$= 2E[x]\sigma_x^2 + 2E[y_f]\sigma_x^2 = 2\bar{z}_f \sigma_x^2$$

$$\mathrm{cov}[z_f^2, z_m^2] = \mathrm{cov}[x^2 + 2xy_f + y_f^2, x^2 + 2xy_m + y_m^2]$$

$$= \mathrm{cov}[x^2, x^2] + \mathrm{cov}[x^2, 2xy_m] + \mathrm{cov}[2xy_f, x^2] + \mathrm{cov}[2xy_f, 2xy_m]$$

$$= (E[x^4] - E[x^2]^2) + 2(E[x^3 y_m] - E[x^2]E[xy_m])$$

$$+ 2(E[x^3 y_f] - E[xy_f]E[x^2]) + 4(E[x^2 y_f y_m] - E[xy_f]E[xy_m])$$

$$= 4E[x]^2\sigma_x^2 + 4E[x]\sigma_x^2 E[y_m] + 4E[x]\sigma_x^2 E[y_f] + 4\sigma_x^2 E[y_f]E[y_m] + 2\sigma_x^4$$

$$= 2\sigma_x^2(2\bar{z}_f \bar{z}_m + \sigma_x^2)$$

Several of the specific calculations immediately above follow from basic probability theory (*e.g.*, https://en.wikipedia.org/wiki/Covariance#Properties), including the general definition of covariance between a pair of random variables X and Y: $\mathrm{cov}(X, Y) = E[XY] - E[X]E[Y]$. Substituting back into eq. (A4), and following some algebra, we obtain:

$$\mathrm{cov}[\ln(W_f), \ln(W_m)] = \sigma_x^2 \frac{2(O_f - \bar{z}_f)(O_m - \bar{z}_m) + \sigma_x^2}{2\omega_f^2 \omega_m^2} \quad \text{(A5)},$$

which is equivalent to eq. (3) in the main text.

Finally, using eqs. (A3) and (A5), the cross-sex genetic correlation for fitness, measured on logarithmic scale, becomes:

$$r_W^{\mathrm{fm}} = \frac{\mathrm{cov}\left[\ln\left(W_f\right), \ln(W_m)\right]}{\sqrt{\mathrm{var}\left[\ln\left(W_f\right)\right]\mathrm{var}\left[\ln\left(W_f\right)\right]}} \qquad \text{(A6)},$$

$$= \frac{\sigma_x^2}{\sigma_f \sigma_m} \frac{2\left(O_f - \bar{z}_f\right)(O_m - \bar{z}_m) + \sigma_x^2}{\sqrt{\left(2\left(O_f - \bar{z}_f\right)^2 + \sigma_f^2\right)(2(O_m - \bar{z}_m)^2 + \sigma_m^2)}}$$

which is equivalent to eq. (5) in the main text.

Calculations in standard (*i.e.*, non-logarithmic) scale

When fitness is Gaussian, the calculations become more cumbersome in the standard scale (*i.e.*, non-logarithmic) compared to logarithmic scale (see above). Nevertheless, we can show that results in the logarithmic scale are roughly equivalent to those in the standard scale as long as both sexes are sufficiently well adapted that their fitness surfaces are concave within the region of phenotypic space occupied by members of the population. Indeed, this condition is typically invoked as the justification for using the Gaussian fitness surface in the first place (*e.g.*, Lande 1976; Manna et al. 2011).

The exponential term in the Gaussian fitness function can be expanded as a power series:

$$W_j = C_j \exp\left(-\frac{\left(O_j - z_j\right)^2}{2\omega_j^2}\right)$$

$$= C_j\left(1 - \frac{\left(O_j - z_j\right)^2}{2\omega_j^2} + \frac{1}{2!}\left[\frac{\left(O_j - z_j\right)^2}{2\omega_j^2}\right]^2 - \frac{1}{3!}\left[\frac{\left(O_j - z_j\right)^2}{2\omega_j^2}\right]^3 + \cdots\right)$$

When members of each sex are not strongly displaced from their optimum (*i.e.*, $\frac{\left(O_j - z_j\right)^2}{2\omega_j^2} \ll 1$), then the fitness surface is roughly quadratic with respect to the trait variation present within the population. When the fitness surface is quadratic, or approximately so, then our fitness function is:

$$W_j \approx C_j\left(1 - \frac{(O_j - z_j)^2}{2\omega_j^2}\right)$$

The variance in the jth sex becomes:

$$\mathrm{var}(W_j) \approx C_j^2 \mathrm{var}\left[\frac{(O_j - z_j)^2}{2\omega_j^2}\right] = C_j^2 \mathrm{var}[\ln(W_j)]$$

The cross-sex covariance becomes:

$$\mathrm{cov}(W_f, W_m) \approx C_f C_m \mathrm{cov}\left[\frac{(O_f - z_f)^2}{2\omega_f^2}, \frac{(O_m - z_m)^2}{2\omega_m^2}\right] = C_f C_m \mathrm{cov}[\ln(W_f), \ln(W_m)]$$

And, finally, the cross-sex genetic correlation for fitness in standard scale becomes:

$$r_W^{\mathrm{fm}} = \frac{\mathrm{cov}(W_f, W_m)}{\sqrt{\mathrm{var}(W_f)\mathrm{var}(W_m)}} \approx \frac{\mathrm{cov}[\ln(W_f), \ln(W_m)]}{\sqrt{\mathrm{var}[\ln(W_j)]\mathrm{var}[\ln(W_j)]}}$$

In other words, the cross-sex genetic correlation for fitness, as calculated on the standard scale, is roughly equivalent to the logarithmic scale result (eq. (A6), above).

Gaussian functions may no longer be biologically justified for severely maladapted populations or individuals that are far from optimum (as discussed elsewhere: Manna et al. 2011). Nevertheless, it is worth considering how much results for the standard scale diverge from those in the logarithmic scale in cases where fitness functions are Gaussian and both sexes are strongly displaced from their optima. Simulated fitness variances and covariances in both scales show that the cross-sex genetic correlation for fitness in the standard scale becomes increasingly sensitive to the strength of stabilizing selection on the trait as each sex becomes further displaced from its optimum (see Fig. S1, below, where r_W^{fm} values in standard scale depend, in part, on the "curvature" of the Gaussian fitness surface, represented by ω_f and ω_m). When stabilizing selection for the trait is weak or modest ($\omega_j \gg 1$), the exact analytical predictions on logarithmic scale (eq. (4) from the main text) closely match predictions for the cross-sex fitness correlation in the standard scale. With strong stabilizing

selection on the trait and pronounced deviations of each sex from its optimum (*i.e.*, small values of ω_j coupled with large magnitudes of d_m and d_f; see the bottom right-hand panel of Fig. S1), eq. (4) predicts the sign of r_W^{fm} in the standard scale, but is no longer an accurate approximation for its magnitude (see the bottom right panel of Fig. S1).

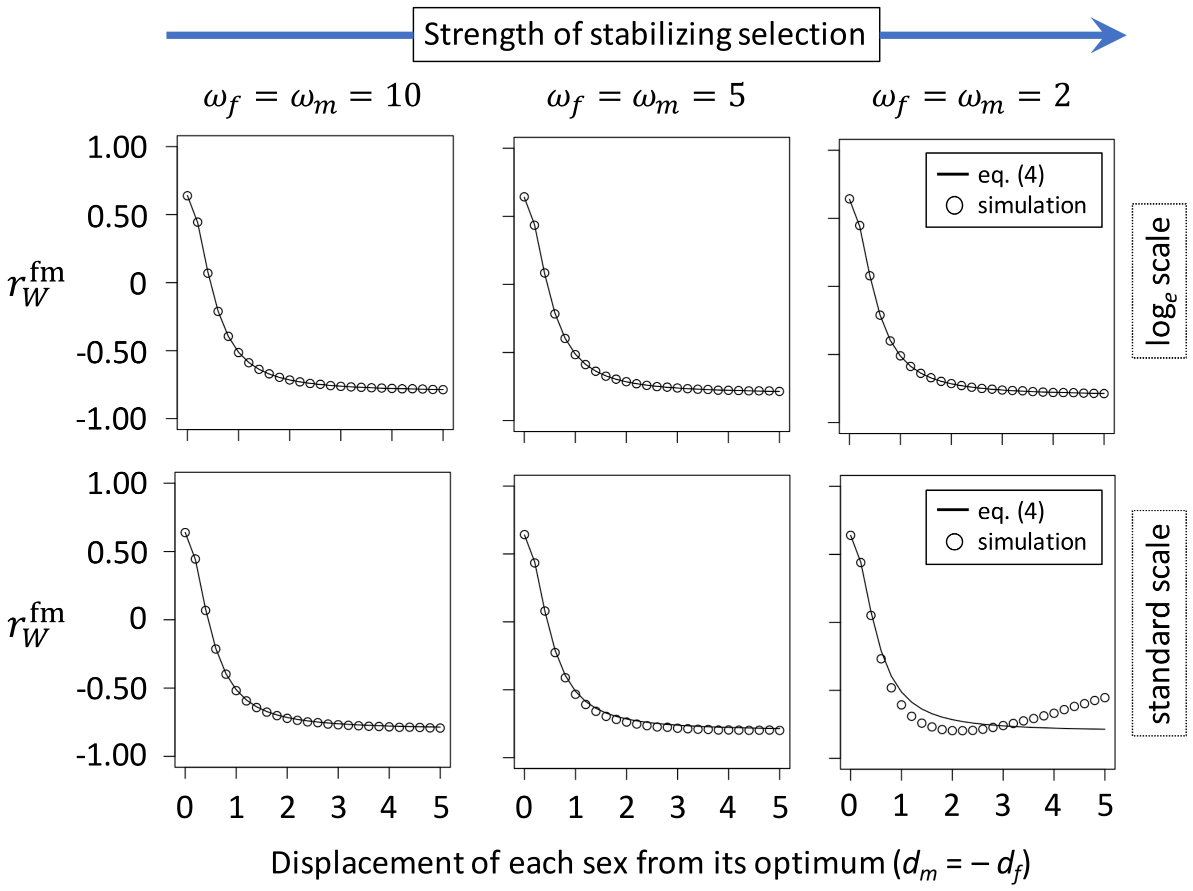

Figure S1. Predictions of the cross-sex genetic correlation for fitness based on the Gaussian fitness model. Calculations in logarithmic scale (log$_e$) are exact for the model, and always align well with simulated data (top panels). Predictions for each of the two scales align strongly as long as the population is reasonably well adapted to its environment, *i.e.*: the displacement of each sex from its optimum (depicted by d_m and d_f) is not too severe relative to the curvature of the fitness surface (depicted by ω_f and ω_m). Results use the parameters $r_z = 0.8$ and $\sigma_f^2 = \sigma_m^2 = 0.5$.

Appendix II. Derivation of the multivariate model

The general expression for fitness, allowing for differences in variance and stabilizing selection among traits, is:

$$\ln(W_j) = \ln(C_j) - \frac{1}{2}\sum_{i=1}^{n}\frac{\left(O_{i,j} - z_{i,j}\right)^2}{\omega_{i,j}^2}$$

Assuming that each of the trait axes varies independently of the others, the fitness variance in the *j*th sex and fitness covariance between sexes (respectively) will be:

$$\mathrm{var}[\ln(W_j)] = \frac{1}{4}\mathrm{var}\left(\sum_{i=1}^{n}\frac{\left(O_{i,j} - z_{i,j}\right)^2}{\omega_{i,j}^2}\right) = \frac{1}{4}\sum_{i=1}^{n}\frac{1}{\omega_{i,j}^4}\mathrm{var}\left(\left(O_{i,j} - z_{i,j}\right)^2\right)$$

$$= \frac{1}{2}\sum_{i=1}^{n}\frac{\sigma_{i,j}^2\left(2d_{i,j}^2 + \sigma_{i,j}^2\right)}{\omega_{i,j}^4}$$

and

$$\mathrm{cov}[\ln(W_m), \ln(W_f)] = \frac{1}{4}\mathrm{cov}\left[\sum_{i=1}^{n}\frac{\left(O_{i,f} - z_{i,f}\right)^2}{\omega_{i,f}^2}, \sum_{i=1}^{n}\frac{\left(O_{i,m} - z_{i,m}\right)^2}{\omega_{i,m}^2}\right]$$

$$= \frac{1}{4}\sum_{i=1}^{n}\frac{\mathrm{cov}\left[\left(O_{i,f} - z_{i,f}\right)^2, \left(O_{i,m} - z_{i,m}\right)^2\right]}{\omega_{i,f}^2\omega_{i,m}^2} = \frac{1}{2}\sum_{i=1}^{n}\frac{\sigma_{i,x}^2\left(2d_{i,f}d_{i,m} + \sigma_{i,x}^2\right)}{\omega_{i,f}^2\omega_{i,m}^2}$$

where $d_{i,j} = O_{i,j} - \bar{z}_{i,j}$. From these expressions, the cross-sex additive genetic correlation for fitness becomes:

$$r_{\mathrm{W}}^{\mathrm{fm}} = \frac{\sum_{i=1}^{n}\frac{\sigma_{i,x}^2\left(2d_{i,f}d_{i,m} + \sigma_{i,x}^2\right)}{\omega_{i,f}^2\omega_{i,m}^2}}{\sqrt{\left(\sum_{i=1}^{n}\frac{\sigma_{i,f}^2\left(2d_{i,f}^2 + \sigma_{i,f}^2\right)}{\omega_{i,f}^4}\right)\left(\sum_{i=1}^{n}\frac{\sigma_{i,m}^2\left(2d_{i,m}^2 + \sigma_{i,m}^2\right)}{\omega_{i,m}^4}\right)}}$$

as presented in the main text.

In a well-adapted population at equilibrium ($d_{i,j} = 0$), we have:

$$r_{\mathrm{W}}^{\mathrm{fm}} = \frac{\sum_{i=1}^{n} \frac{\left(r_{i,z}\sigma_{i,f}\sigma_{i,m}\right)^2}{\omega_{i,f}^2\omega_{i,m}^2}}{\sqrt{\left(\sum_{i=1}^{n}\left(\frac{\sigma_{i,f}^2}{\omega_{i,f}^2}\right)^2\right)\left(\sum_{i=1}^{n}\left(\frac{\sigma_{i,m}^2}{\omega_{i,m}^2}\right)^2\right)}}$$

where $r_{i,z} = \sigma_{i,x}^2\left(\sigma_{i,f}\sigma_{i,m}\right)^{-1}$. Assuming that cross-sex genetic correlations are constant across traits ($r_z = r_{i,z}$), the last expression reduces to:

$$r_{\mathrm{W}}^{\mathrm{fm}} = r_z^2 \frac{\sum_{i=1}^{n} \frac{\sigma_{i,f}^2}{\omega_{i,f}^2}\frac{\sigma_{i,m}^2}{\omega_{i,m}^2}}{\sqrt{\left(\sum_{i=1}^{n}\left(\frac{\sigma_{i,f}^2}{\omega_{i,f}^2}\right)^2\right)\left(\sum_{i=1}^{n}\left(\frac{\sigma_{i,m}^2}{\omega_{i,m}^2}\right)^2\right)}} = r_z^2\cos(\theta_0) \le r_z^2$$

where θ_0 is the angle between female and male vectors of $\sigma_{i,j}^2/\omega_{i,j}^2$ across the set of traits. The final inequality at the right reflects the mathematical constraint, $\cos(\theta_0) \le 1$. When the relative strengths of selection on each trait are the same in each sex, so that $\frac{\sigma_{i,f}^2}{\omega_{i,f}^2} = c\frac{\sigma_{i,m}^2}{\omega_{i,m}^2}$ where *c* is a constant, then $\cos(\theta_0) = 1$, and therefore, $r_{\mathrm{W}}^{\mathrm{fm}} = r_z^2$. When the relative strengths of selection on each trait differs between sexes, then $\cos(\theta_0) < 1$ and $r_{\mathrm{W}}^{\mathrm{fm}}$ will be further depressed relative to r_z.

In a poorly-adapted population sufficiently far from equilibrium ($\left|d_{i,f}d_{i,m}\right| \gg \sigma_{i,x}^2$, $\left|d_{i,j}\right| \gg \sigma_{i,j}$), we have:

$$r_{\mathrm{W}}^{\mathrm{fm}} \approx \frac{\sum_{i=1}^{n} r_{i,z}\frac{\sigma_{i,f}d_{i,f}\sigma_{i,m}d_{i,m}}{\omega_{i,f}^2\omega_{i,m}^2}}{\sqrt{\left(\sum_{i=1}^{n}\left(\frac{\sigma_{i,f}d_{i,f}}{\omega_{i,f}^2}\right)^2\right)\left(\sum_{i=1}^{n}\left(\frac{\sigma_{i,m}d_{i,m}}{\omega_{i,m}^2}\right)^2\right)}}$$

Assuming that cross-sex genetic correlations are constant across traits ($r_z = r_{i,z}$), the approximation reduces to:

$$r_{\mathrm{W}}^{\mathrm{fm}} = r_z\cos(\theta_d)$$

where θ_d is the angle between the vector of $\sigma_{i,f} d_{i,f} / \omega_{i,f}^2$ for females and the vector of $\sigma_{i,m} d_{i,m} / \omega_{i,m}^2$ for males.
